# The Molecular Circadian Clock and Alcohol-Induced Liver Injury

**DOI:** 10.3390/biom5042504

**Published:** 2015-10-14

**Authors:** Uduak S. Udoh, Jennifer A. Valcin, Karen L. Gamble, Shannon M. Bailey

**Affiliations:** 1 Department of Pathology, Division of Molecular and Cellular Pathology, University of Alabama at Birmingham, Birmingham, AL 35233, USA; E-Mails: uduaksu@gmail.com (U.S.U.); javalcin@uab.edu (J.A.V.); 2 Department of Psychiatry, Division of Behavioral Neurobiology, University of Alabama at Birmingham, Birmingham, AL 35233, USA; E-Mail: klgamble@uab.edu (K.L.G.)

**Keywords:** alcohol, ethanol, liver, hepatotoxicity, steatosis, circadian, molecular clock, circadian desynchrony

## Abstract

Emerging evidence from both experimental animal studies and clinical human investigations demonstrates strong connections among circadian processes, alcohol use, and alcohol-induced tissue injury. Components of the circadian clock have been shown to influence the pathophysiological effects of alcohol. Conversely, alcohol may alter the expression of circadian clock genes and the rhythmic behavioral and metabolic processes they regulate. Therefore, we propose that alcohol-mediated disruption in circadian rhythms likely underpins many adverse health effects of alcohol that cut across multiple organ systems. In this review, we provide an overview of the circadian clock mechanism and showcase results from new studies in the alcohol field implicating the circadian clock as a key target of alcohol action and toxicity in the liver. We discuss various molecular events through which alcohol may work to negatively impact circadian clock-mediated processes in the liver, and contribute to tissue pathology. Illuminating the mechanistic connections between the circadian clock and alcohol will be critical to the development of new preventative and pharmacological treatments for alcohol use disorders and alcohol-mediated organ diseases.

## 1. Introduction

Excessive alcohol use remains a top ten cause of preventable death in the US [1]. Statistics show that alcohol use contributes to almost 90,000 deaths and over 2.5 million years of potential life lost for each year in the US from 2006–2010 [2,3]. The economic costs associated with alcohol use are also staggering with annual costs estimated at $ 224 billion [4]. Heavy alcohol consumption can cause numerous chronic diseases including liver and heart disease, cancer, neurological disorders, mental health problems, and alcohol dependence. About half of Americans age 18 or older report being current alcohol drinkers, with approximately 5% reporting as being heavy drinkers (*i.e.*, >2 drinks/day for men and >1 drink/day for women) [5]. It is also estimated that 30% of adults experience alcohol use disorders in their lifetime, with 18% from alcohol abuse and 12% from alcohol dependence [6]. Binge drinking has also been recently identified as a significant risk factor for disease. According to the National Institute on Alcohol Abuse and Alcoholism, binge drinking is defined as a drinking pattern that brings an individual’s blood alcohol concentration up to 0.08 g/dL within a short period of time (*i.e.*, within 2 h), and is typically achieved by consuming four drinks for women and up to five drinks for men. A 2012 national survey reported that 25% of people age 18 or older engage in binge drinking each month [7].

Alcoholic liver disease (ALD) is a significant cause of morbidity and mortality from heavy and binge alcohol use. It is the number one cause of death from chronic alcohol consumption. Two million Americans are afflicted with various stages of ALD causing an estimated 15,000–20,000 deaths per year [8]. Regrettably, there are few successful treatments for ALD. These dire statistics illustrate the need to improve scientific and medical knowledge of the underlying causes of ALD so that effective therapies can be developed for treatment. Collectively, excessive alcohol use places a substantial economic, societal, and health burden on the US.

ALD is a spectrum of pathologies ranging from steatosis (*i.e.*, fatty liver) to the more severe conditions of alcoholic steatohepatitis, fibrosis, cirrhosis, and hepatocellular carcinoma. While it has long been believed that the severity of ALD is dependent on the dose and duration of alcohol consumption, it has become clear that, like many other diseases, the pathology of ALD is complex and a product of multiple gene-environment-metabolic interactions. This concept is highlighted by the observation that only 25% of heavy drinkers develop alcoholic steatohepatitis with less than 10% progressing to cirrhosis [9]. Thus, it is readily apparent that in addition to dose and duration other factors are likely required for severe liver pathology to occur in the chronic alcohol consumer. This review article will consider the potential importance of the molecular circadian clock in ALD. Recent studies provide compelling evidence that disruption in circadian clock function and/or circadian misalignment (*a.k.a.*, desynchrony) may be another important risk factor to consider in the complex pathobiology of alcohol-related tissue injury [10,11,12,13,14]. Herein, we will describe the components that comprise the molecular circadian clock, discuss certain mechanisms proposed to regulate clock function, and provide an overview of new work showing the likely role of circadian clock disruption in alcohol hepatotoxicity. We also propose a hypothetical mechanism by which chronic alcohol may alter circadian clock function in the liver and contribute to ALD.

## 2. Overview of Alcoholic Liver Disease Mechanisms

**Figure 1:**
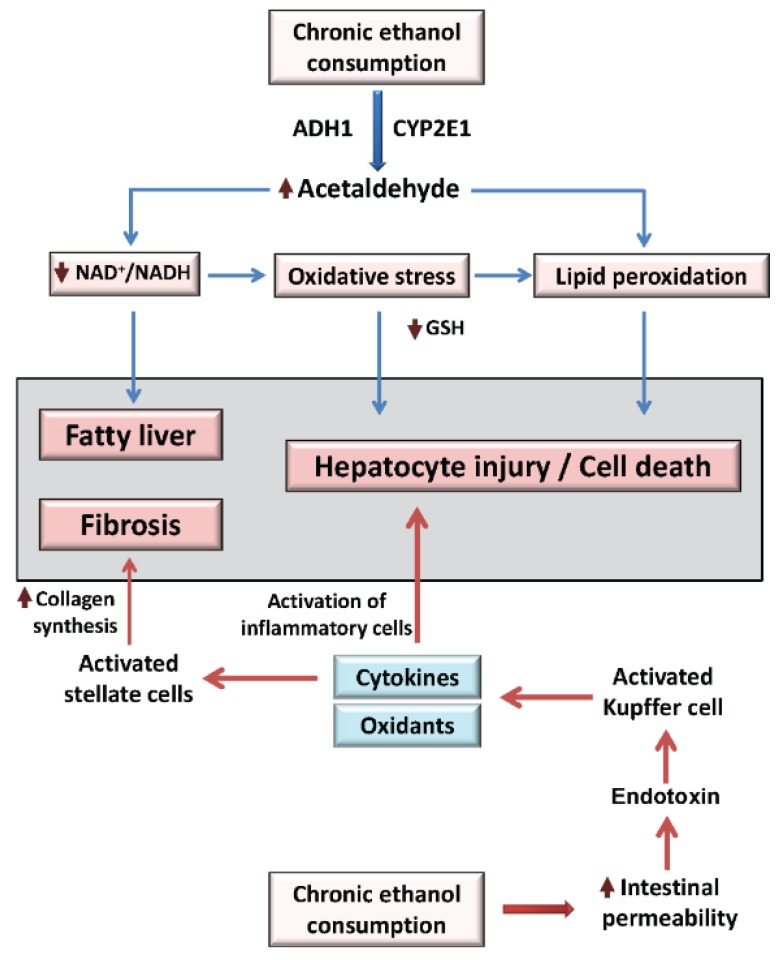
Pathogenic pathways of alcoholic liver disease. Alcohol is oxidized to acetaldehyde in the liver by alcohol dehydrogenase 1 (ADH1) and cytochrome P450 2E1 (CYP2E1). Alcohol metabolism leads to redox imbalance (↓NAD^+^/NADH), accumulation of the highly toxic acetaldehyde, and in turn alterations in lipid metabolic pathways, adduct formation, increased oxidative stress, glutathione (GSH) depletion, mitochondrial damage, and consequently liver injury. Chronic alcohol consumption also increases gut permeability, thereby promoting endotoxin leakage into the portal vein circulation. Gut-derived endotoxin in the liver activates Kupffer cells, inducing the release of pro-inflammatory cytokines such as TNF-α, which in turn activate hepatic stellate cells to produce TGF-β and collagen, leading to fibrosis. Additionally, activated Kupffer cells produce reactive oxidants through NADPH oxidase further exacerbating oxidative stress.

Chronic alcohol consumption dramatically alters liver function at multiple levels including histologic, metabolic, genomic, and proteomic levels, with each of these implicated in the disease process (Figure 1). All cell types in the liver (e.g., hepatocyte, Kupffer cells, stellate cells, and sinusoidal endothelial cells) are also implicated in the etiology of ALD, as well as a number of different types of infiltrating immune cells including monocytes, macrophages and neutrophils [15,16]. ALD may also be considered a multi-organ disease, as increased intestinal leakage of endotoxin into the portal circulation and disrupted adipose-mediated cytokine circuits negatively impact liver injury in the chronic alcohol consumer [17,18,19]. A large number of putative metabolic and molecular alterations occurring in the early steatosis stage contribute to the development of ALD, including triglyceride accumulation, glycogen depletion, redox imbalances, mitochondrial dysfunction, lipid peroxidation, oxidative stress, protein adduct formation, proteasome defects, and inflammation [16,20,21]. Importantly, increased fat (e.g., triglyceride and free fatty acid species) and decreased glycogen content are proposed to render hepatocytes more vulnerable to additional insults and metabolic stressors leading to hepatocyte death, a recognized critical first hit for ALD progression [22].

Multiple studies show that alcohol-dependent dysregulation of signaling pathways (e.g., SREBP-1, PPARα/γ, AMPK) leads to increased lipogenesis, decreased fatty acid oxidation, excess hepatic lipid accumulation, and the development of alcoholic steatosis [23]. Critical to these mechanisms of ALD are reports showing that some components of these metabolic pathways exhibit time-of-day alterations at multiple levels; e.g., gene, protein, and activity [24,25,26,27,28,29]. There is also a growing appreciation that the molecular circadian “clock”, an intrinsic cellular mechanism, can markedly affect the responsiveness of peripheral tissues, like the heart and liver, to various insults and stressors in a time-of-day dependent manner. While still a new area of investigation, the circadian clock is now being examined within the context of alcohol-induced tissue pathologies, with several laboratories showing alcohol-mediated alterations to the clock in liver and intestine [10,11,12,13,14]. Before discussing this new work in alcohol field, the following sections will provide an overview of the molecular circadian clock mechanism and regulation of the clock by multiple inputs.

## 3. The Molecular Circadian Clock and Metabolism

Many metabolic processes oscillate during the day, enabling organisms/tissues/cells to remain in synchrony with their environment. For example, 5%–20% of the liver transcriptome and proteome varies during the day affecting many metabolic and signaling networks [29,30,31]. Daily rhythms in metabolism tend to align with, but are not dependent upon, sleep/wake and fasting/feeding cycles. In fact, many metabolic rhythms are partially controlled by an intrinsic circadian oscillator or “molecular clock”. Circadian clocks allow cells to rapidly respond to environmental stimuli or stress by adapting metabolism in a temporally appropriate manner [32,33]. The importance of clocks in regulating metabolism and maintaining cellular health is exemplified by the fact that mouse models in which clocks are genetically altered (e.g., mutated and ablated) throughout the body exhibit profound alterations in behavior, metabolism, and immune responses, leading to various pathologies [34]. Similarly, humans with disrupted circadian systems (e.g., night-shift workers) are at higher risk for cardiometabolic diseases, such as dyslipidemia, obesity, type 2 diabetes, and hypertension [35,36]. Thus, it has been hypothesized that the circadian clock is essential for maintaining cellular homeostasis and whole body health.

## 4. Core Components of the Molecular Circadian Clock

**Figure 2:**
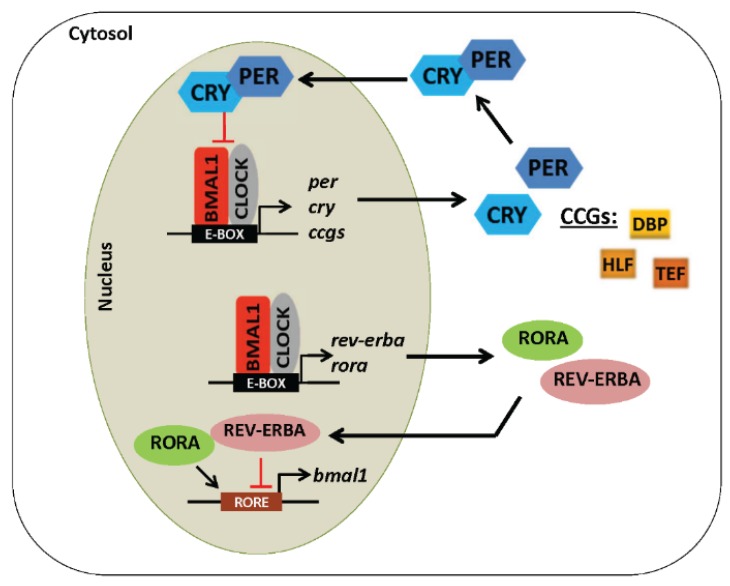
The molecular circadian clock mechanism. The core circadian clock machinery is made up of transcriptional-translational positive-negative feedback loops that mediate ~24 h autonomous rhythms in clock genes and various clock-controlled metabolic processes.

The molecular circadian clock relies on a transcriptional-translational feedback loop comprised of the transcription factors, circadian locomotor output cycles kaput (CLOCK) and brain and muscle aryl hydrocarbon nuclear translocator-like 1 (BMAL1). CLOCK and BMAL1 serve as positive regulators of the molecular clock mechanism. In the nucleus, CLOCK and BMAL1 heterodimerize and form a CLOCK-BMAL1 complex, which binds to the E-box element in the promoters of target genes (see review [37]). Importantly, CLOCK-BMAL1 activate the expression of the negative regulators of the molecular clock, Period 1, 2, and 3 (*per1*, *per2*, and *per3*) and Cryptochrome 1 and 2 (*cry1* and *cry2*). A scheme depicting the molecular clock mechanism is provided in Figure 2.

PER and CRY proteins heterodimerize and translocate to the nucleus where they inhibit their own transcription by interfering with CLOCK-BMAL1 activity. CLOCK-BMAL1 also promote the transcription of many non-core clock controlled genes, such as the transcription factors albumin D-site binding protein (*dbp*), hepatic leukemia factor (*hlf*), and thyrotroph embryonic factor (*tef*), and various other metabolic genes. Another separate, but very important, interacting transcriptional-translational feedback loop, functions to stabilize and reinforce the oscillations driven by the core clock loop. In this second feedback loop, the orphan nuclear receptors, retinoid acid receptor-related orphan receptor (ROR) alpha, beta, and gamma, and REV-ERB alpha and beta (also known as NR1D1 and NR1D2, or nuclear receptor subfamily 1, group D, member 1 or 2), bind to ROR response elements (RORE) to activate and inactivate *bmal1* expression, respectively [38,39,40] (Figure 2). Jointly, these positive-negative feedback loops make up the molecular circadian oscillator, and their components oscillate with a period of approximately 24 h. As will be discussed later in this review, post-translational modifications (PTMs) of clock proteins provides an additional layer of temporal control to the clock by regulating protein-protein interactions, protein degradation by the proteasome, DNA binding activity, cellular localization, and nuclear translocation [41].

The primary, central circadian clock is located within the suprachiasmatic nucleus (SCN) of the hypothalamus, while peripheral clocks are found within non-SCN cells of the organism, including other regions of the CNS and other organ systems in the body [42]. Zeitgebers (“time-givers”) are factors which reset (or entrain) central and peripheral circadian clocks. The SCN is primarily reset by light (via electrical signals transmitted along the retino-hypothalamic tract), while peripheral clocks are synchronized by the central clock via modulation of neuro-humoral stimuli, either directly (e.g., through innervations between the SCN and specific peripheral tissues) and/or indirectly (e.g., through alterations in feeding behavior, release of feeding-responsive hormones, paracrine signals, and/or diurnal variations in temperature) [42,43,44]. This normal synchrony (*i.e.*, special timing relationship) between central and peripheral clocks can easily be lost due to environmental and/or behavioral interventions. For example, forcing rodents to eat during the inactive (sleep) phase causes peripheral clocks to phase shift by 12 h, while the central SCN clock is unaffected [45]. This desynchrony is associated with weight gain in mice [46]. An emerging hypothesis in the alcohol field is that chronic alcohol consumption causes desynchrony between the SCN clock and the liver clock and/or other peripheral tissue clocks, and this disruption contributes to tissue pathology.

## 5. Regulation of the Circadian Clock

The circadian clock confines different metabolic programs to metabolically necessary times of the day. This is accomplished by coordinated cycling of clock activators and repressors as shown in Figure 2. As a result, the timing of energy metabolism (storage and utilization) is synchronized to nutrient intake in order to ensure proper cellular physiology. Importantly, the liver (and other peripheral organs) can be thought of as a completely different organ at different times of the day. It is easy to envision then that disruption of the circadian clock by environmental insults and metabolic stressors (e.g., alcohol) might result in misalignment of circadian regulated metabolic pathways, leading to metabolic disorders [45,47,48]. This concept is supported by genetic studies where mutation or knockout of clock components perturbs rhythmic expression of metabolic genes [49,50]. As mentioned earlier, peripheral tissue clocks can be synchronized by signals (neural and hormonal) originating from the central clock in the SCN [51]. However, peripheral tissue clocks are also modulated by SCN-independent cues; e.g., food intake [52,53] and metabolite fluxes [54]. This allows for dynamic shifts in rhythmic metabolic processes and shows that metabolism strongly influences function and timing (phase) of the clock. This is especially true for the liver. In the following sections, we will highlight the role of cellular redox status, post-translational modifications of proteins, and various post-transcriptional mechanisms on circadian clock function.

### 5.1. Cellular Redox Status and the Clock

A growing body of evidence demonstrates that there is a strong interaction between the circadian clock and metabolism. For instance, changes in circadian clock oscillations are observed in metabolic diseases like diabetes and obesity [55,56,57]. Conversely, alterations in cellular metabolism influence timing of the circadian clock. Some of the earliest work demonstrating that metabolism can impact the functional activity of the circadian clock focused on the role of the cellular redox state. Support for a link between cellular redox metabolism and clock regulation was first provided by studies showing that DNA binding of CLOCK-BMAL1 [or neuronal PAS domain protein 2 (NPAS2)-BMAL1] can be modulated by levels of pyridine nucleotides [58]. For example, high levels of NAD(P)H increases NPAS2-BMAL1 DNA binding, whereas NAD(P)^+^ decreases DNA binding in an *in vitro* system with purified proteins. Rutter *et al.* [58] also showed cells transfected with expression vectors encoding NPAS2 and BMAL1 accumulated lactate hydrogenase (*ldha*) mRNA, demonstrating *ldha* as a clock gene target. LDH helps to maintain the cytosolic redox state, as it’s co-factor for the reaction converting lactate to pyruvate (and *vice versa*) is NAD^+^ (and NADH). Therefore, it is highly probable that *ldha*, a target gene of NPAS2/CLOCK-BMAL1, has the ability to feedback and regulate clock function by changing the cellular redox state. As the ratio of NAD^+^ cofactors is considered as a “read-out” of metabolism, it is highly probable that alterations in redox state affect activity of cellular clocks *in vivo*; however, whether this level of regulation occurs *in vivo* is unknown. The importance of cellular redox in clock control is also exemplified by observations that levels of NAD^+^ exhibit daily rhythms in the liver due to clock-dependent regulation of nicotinamide phosphoribosyltransferase (NAMPT), the rate-limiting enzyme in the NAD^+^ salvage pathway [59,60]. Notably, alcohol is metabolized primarily in the liver resulting in a decreased NAD^+^/NADH ratio [61]. Based on this background, it is predicted that alcohol consumption would alter circadian clock rhythms in the liver as a result of ethanol-mediated alteration in the cellular redox state [62]. In line with this prediction, data from our laboratory data shows, for the first time, that chronic alcohol ingestion significantly alters the phase, amplitude, and mean expression of liver clock gene rhythms [10]. Others have replicated these results [12]. Taken together, these studies support the concept that transcriptional activity of the circadian clock can be modulated by changes in pyridine nucleotides. A hypothetical mechanism for how alcohol-induced alterations in cellular redox state might affect the circadian clock in the liver is proposed later in this review article.

### 5.2. Post-Translational Modifications and the Clock

**Figure 3:**
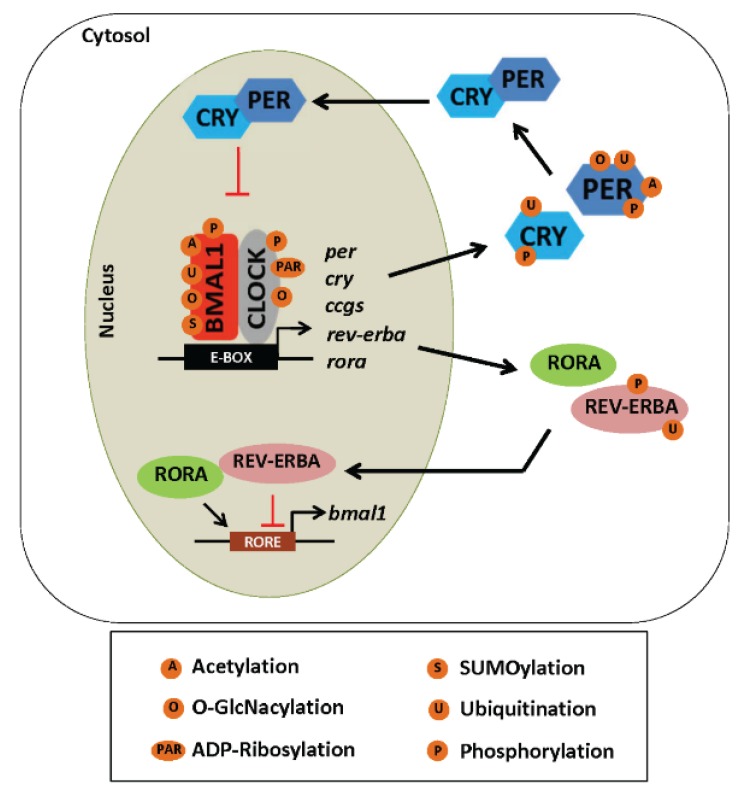
Post-translational modifications of circadian clock proteins. This scheme summarizes some of the various post-translational modifications (PTMs) that have been documented to occur on circadian clock proteins. Descriptions of these modifications and insights regarding how these various PTMs affect clock protein function, stability, and subcellular localization is provided in the text along with relevant references.

The activity of the molecular clock oscillator can be fine-tuned by several key post-translational modifications (PTMs), including phosphorylation, acetylation, and ADP-ribosylation. Some of these PTMs are important for regulating the circadian clock cycle by inducing or repressing transcriptional activation, whereas others are involved in controlling intracellular localization of clock components [63]. PTMs also play a key role in mediating how various environmental entrainment factors synchronize an organism’s circadian clock with its environment. For example, phosphorylation of different clock components plays a significant role in maintaining the “speed” of the clock to the 24 h day [64]. The following sections provide brief overviews of several PTMs shown to influence clock function in mammalian cells. A summary of these PTMs is provided in Figure 3.

#### 5.2.1. Phosphorylation

The activity and subcellular localization of several core clock proteins is modulated by phosphorylation. For example, the “speed” or timing of the clock is believed to be regulated by phosphorylation of PER proteins by casein kinase 1 delta/epsilon (CSNK1D/E) in mammals and it’s homolog in *Drosophila* called DOUBLETIME [65,66,67]. The subcellular localization of PER is also dependent on phosphorylation by CSNK1E, and likely affects PER-mediated transcriptional repression of the clock and PER degradation [68,69,70]. In contrast, phosphorylation of CRY1 and 2 proteins stimulates their interaction and enables their subsequent degradation by the ubiquitin ligase FXBL3 [71,72,73]. This occurs through the concerted action of AMPK, a kinase believed to act as a clock resetting signal [74]. The core clock protein BMAL1 is also phosphorylated by CSNK1E, an event leading to activation of BMAL1-mediated transcription [75], whereas phosphorylation of BMAL1 by mitogen activated protein kinase (MAPK) inhibits BMAL1-mediated transcription [76]. Another important kinase involved in regulating clock function is the serine/threonine kinase, glycogen synthase kinase 3 beta (GSK3β). GSK3β activity oscillates in the SCN [77] and in some peripheral tissues [78,79]. GSK3β also phosphorylates several clock proteins and regulates the amplitude of clock protein rhythms [80]. Phosphorylation, however, does have different effects on the stability of clock proteins. For example, GSK3β-mediated phosphorylation increases stability of REV-ERBα [81], but induces degradation of CRY2 [82] and BMAL1 [80]. Furthermore, treatment with a pharmacological GSK3 inhibitor lithium induces degradation of REV-ERBα, leading to increased BMAL1 expression [81].

#### 5.2.2. Acetylation

Protein acetylation is a key regulator of clock function in several tissues and cell systems. Importantly, the core clock transcription factor CLOCK has histone acetyltransferase (HAT) function [83], which induces rhythmic acetylation of BMAL1 [84]. This action of CLOCK on BMAL1 is dependent on the formation of the CLOCK-BMAL1 heterodimer. Moreover, acetylation of BMAL1 by CLOCK HAT activity is important for facilitating CRY1-dependent repression of the clock [84], contributing to the negative limb of the circadian feedback loop. Conversely, CLOCK-mediated histone acetylation is involved with transcriptional stimulation of clock-controlled genes [83], thereby contributing to the positive limb of the loop. Deacetylation of BMAL1 also occurs via the action of the NAD^+^-dependent sirtuin 1 (SIRT1) [27]. An important characteristic of this function is that SIRT1 activity is proposed to vary during the day as a function of daily oscillations of its co-factor NAD^+^ [27]. As mentioned earlier, time-of-day dependent variations in NAD^+^ have been shown in several tissues due to oscillations in the expression of the enzyme NAMPT [59,60], the rate-limiting enzyme in the NAD^+^ salvage pathway. This pathway is believed to operate in a circadian-dependent fashion as *nampt* gene expression is clock-regulated [60]. As chronic alcohol consumption has been reported to inhibit SIRT1 in liver [85,86], it is predicted that alcohol-mediated alterations in clock activity may be mediated through a disruption in the balance of the acetylation/deacetylation status of clock proteins. Future studies into the area are warranted and would likely shed light on key mechanistic information regarding the importance of SIRT1 in alcohol-mediated fatty liver disease.

#### 5.2.3. ADP-Ribosylation

Poly ADP-ribose polymerase 1 (PARP-1), an enzyme typically activated by DNA damage and/or oxidative stress, catalyzes the transfer of ADP-ribose units from NAD^+^ to target proteins, including itself, in a process called ADP-ribosylation [87]. Interestingly, ADP-ribosylation oscillates in a circadian manner, with peak activity beginning at the start of the inactive (light) phase in mice [88]. This work also shows that PARP-1 interacts with both BMAL1 and CLOCK, and that PARP-1 ADP-ribosylates CLOCK [88]. This has the effect of modulating the DNA binding activity of the CLOCK-BMAL1 heterodimer; thereby, affecting the positive limb of the circadian feedback loop. Of special interest, is that the ability of PARP-1 and SIRT1 to impact clock function is likely to be interconnected as both enzymes require NAD^+^ for functionality [89]. Thus, activation of PARP-1 would be predicted to decrease SIRT1 activity through depletion of the coenzyme NAD^+^. The impact of chronic alcohol on PARP-1 and SIRT1 within the context of the liver clock will be discussed later.

#### 5.2.4. Ubiquitination

Ubiquitination of clock proteins is important for maintaining protein stability and targeting clock proteins for degradation. Interestingly, combinations of different PTMs regulate clock protein activity; e.g., phosphorylation and ubiquitination are often coupled together. An example of this co-dependence is illustrated for the CRY proteins. While ubiquitin-mediated degradation of CRY proteins is controlled by the F-box-type E3 ligase FBXL3 in the nucleus [71,72,73], this action is dependent on phosphorylation by AMPK [74]. A paralog of FBXL3, known as FBXL21, ubiquitinates CRY proteins; however, this action counteracts the action of FBXL3 and stabilizes CRY proteins [90,91]. Another example is provided with REV-ERB. Ubiquitination of REV-ERB mediates its degradation, whereas REV-ERB is stabilized following phosphorylation by GSK3β [81]. Of note, ubiquitination of BMAL1 coincides with times of high transcriptional activity [92]. Removal of ubiquitin groups (*i.e.*, de-ubiquitination) plays an important role in clock function. The deubiquitinating enzyme ubiquitin-specific protease 2 (USP2) displays rhythmicity in peripheral tissues [93]. For example, USP2 binds directly to PER1 in order to regulate PER1 intracellular localization [94]. Due to the well-established role of ubiquitin-proteasome system inhibition in the pathogenesis of alcoholic liver injury, it is likely that ubiquitination status of clock proteins and/or proteasomal degradation of targeted clock proteins would be severely disrupted in livers of heavy alcohol consumers.

#### 5.2.5. SUMOylation

The process of SUMOylation involves the addition of a small ubiquitin-related modifier (SUMO) protein to lysine residues in target proteins [95]. Within the context of the circadian clock, studies show that timing of peak SUMOylation activity coincides with peak phosphorylation events in BMAL1 and requires a functional CLOCK protein [96]. SUMOylation of BMAL1 is involved in the transcriptional activity of other clock components [96]. For example, the highest levels of SUMOylated BMAL1 are present when mRNA expression of the clock-controlled genes *dbp* and *rev-erb* is highest during the day [92]. Furthermore, ubiquitination of BMAL1 requires that it first be SUMOylated [92], highlighting the importance of these two PTMs in regulation of the circadian clock. While a role of SUMOylation has not been identified in ALD, the ubiquitin-conjugating enzyme 9 that is required for SUMOylation is overexpressed in hepatocellular carcinoma [97] and participates in regulating hepatic lipid metabolism [98] and hepatic inflammation [99]. Thus, SUMOylation of clock proteins may be a previously unrecognized mechanism participating in ALD.

#### 5.2.6. O-GlcNAcylation

A relatively new PTM considered to influence clock function is O-GlcNAcylation, a highly conserved, enzyme-catalyzed PTM involving O-linked β-*N*-acetyl-glucosamine addition to serine/threonine residues by O-GlcNAc transferase (OGT). This PTM is reversed by the removal of O-GlcNAc residues by the enzyme O-GlcNAcase (OGA). Importantly, the enzymes responsible for addition and removal of O-GlcNAc oscillate in a time-of-day dependent manner [100], indicating that this protein modification may be involved with circadian clock regulation. Stability of core clock components BMAL1 and CLOCK are modulated by O-GlcNAcylation. For example, rhythmic O-GlcNAcylation leads to inhibition of protein ubiquitination and subsequent prevention of BMAL1 and CLOCK degradation, thereby promoting the expression of CLOCK-BMAL1 heterodimer target genes *per2* and *cry1* [101]. Thus, there is most likely a prominent role for O-GlcNAcylation in the circadian timing mechanism; however, this has not been investigated within the context of ALD.

### 5.3. Post-Transcriptional Mechanisms and the Clock

In addition to the role protein PTMs play in regulating clock function (discussed above), post-transcriptional mechanisms participate in controlling clock activity. Studies by Takahashi and colleagues show that only 22% of rhythmic mRNA transcripts are the result of *de novo* transcription [102]. Similarly, trans-acting RNA-binding proteins such as heterogenous nuclear ribonucleoproteins bind to cis-acting elements in *per* and *cry* transcripts and function to regulate transcript stability at different phases of the circadian cycle [103,104]. Other RNA-binding proteins also bind to mRNA transcripts of clock and clock-output genes and stimulate translation. For example, cold-inducible RNA-binding protein (CIRBP) interacts with *clock* mRNA, resulting in stabilization of *clock* in the cytoplasm [105]. Low levels of CIRBP correlate with low levels of CLOCK, as well as other circadian components, leading to disruption of the normal circadian clock cycle [105]. Together, these studies strongly support the importance of post-transcriptional mechanisms in regulating the mammalian molecular clock.

Another emerging post-transcriptional mechanism of interest involves regulation of the poly(A) tail length of gene transcripts. Green and colleagues reported that Nocturnin (NOC; *a.k.a.*, carbon catabolite repression 4-like protein or CCRN4l), a proposed circadian deadenylase, catalyzes poly(A) tail shortening, which destabilizes the transcript and reduces translational efficiency [106,107,108]. NOC is believed to control metabolic rhythms of some genes involved in lipid metabolism [109], and to be rhythmically expressed in the liver [110]. Interestingly, we found that chronic alcohol consumption dampens the amplitude of the diurnal rhythm of *noc* mRNA in liver of mice [10], suggesting a possible role of this unique circadian deadenylase in alcohol-mediated steatosis.

The more widely studied and best understood mechanism of post-transcriptional regulation to date involves the role of microRNAs (*a.k.a.*, miRNAs or miRs). These single stranded, short, noncoding RNAs (approximately 22 nucleotides in length) have the ability to negatively regulate gene expression by inhibiting protein synthesis through translational repression and degradation of their miRNA-specific targets. New work in the circadian field using genome-wide based approaches has shown that some miRNAs are involved in regulating expression of core clock components [111,112]. For example, studies indicate that miR-219 may control circadian clock speed and timing as silencing miR-219 expression in the SCN lengthens the circadian rhythm period [113]. Moreover, miR-132 are involved in photic regulation of the circadian clock in the SCN [113]. Studies have also identified potential roles of miRNA in regulating the expression of various clock genes. For example, *in vitro* studies using luciferase-based reporter systems in HEK293 cells found that transfection with pre-miR over-expression constructs of miR-494 and miR-142-3p decreases *bmal1* 3'UTR activity [114]. The mammalian *per* genes also contain targets for miRNAs. Nagel *et al.* [115] using a forward genetics screening approach, in combination with a variety of luciferase-based transfection studies, showed that the miRNA-192/194 gene cluster negatively regulates expression of *per1-3*. Overexpression of these miRNAs caused a decrease in circadian period length, possibly through dampening *per* expression. Studies by Chen *et al.* [116] using dicer-deficient (and thereby miRNA deficient) cells and mice showed that the circadian period is decreased by approximately 2 h and linked to an increased rate in PER1-2 translational in the dicer-deficient *vs.* wild-type cells. In these studies, changes in *per* expression were associated with three miRNAs, miR-24, miR-29a, and miR-30a [116]. Thus, growing evidence supports a role of miRNA in regulating the circadian clock.

Within the context of circadian regulation of liver metabolism, several candidate miRNAs have been identified. For example, Gatfield *et al.* [117] identified miR-122 as a candidate for regulating circadian gene expression in the liver. They found that precursor and primary transcripts of miR-122 display circadian rhythmicity with expression peaking in the early morning. However, in liver of REV-ERB*α* knockout mice, they observed that miR-122 transcripts were constitutively elevated and non-cycling, indicating that REV-ERBα may drive transcription of miR-122. Furthermore, a relationship between miR-122 and expression of cholesterol and lipid metabolism genes was found; e.g., expression of *ppar*β*/*δ and *smarcd1/baf60a*, were highly sensitive to miR-122 dependent regulation [117]. Interestingly, NOC has also been identified as a direct target of miR-122 [110]. Knockdown of miR-122 in liver results in an increase in the amplitude of *noc* rhythms, with increased expression at night [110]. Furthermore, mice deficient in *noc* have impairments in lipid homeostasis and glucose responsiveness [118,119]. Taken together, miR-122-dependent regulation of *noc* expression may be an important link between miRNAs, hepatic lipid metabolism, and the circadian clock.

#### MicroRNAs and Alcoholic Liver Disease

MicroRNAs have also been implicated in pathobiology of ALD. For example, expression profiling showed significant alterations (up and down-regulation) in several miRNAs from various experimental murine models of chronic alcohol consumption (see reviews [120,121]). Studies from You and colleagues show that chronic ethanol exposure (in cells and liver) increases levels of miR-217, which dysregulates the function of lipin-1, a key player in lipid metabolism, by sequestering it into the cytosol [86]. Alcohol also causes a miR-217-dependent loss of function of *sirt1* in hepatocytes [86] and Kupffer cells [122]; thus, supporting a link between miR-217 and alcohol-induced hepatic steatosis and inflammation. Similarly, Szabo and colleagues have reported that chronic alcohol consumption alters the expression of various miRNAs [123,124]. For example, miR-122, an abundant miRNA in hepatocytes [125,126], might be a unique biomarker of liver disease, as increased levels of circulating miR-122 are present in conditions of viral, drug, and alcohol-induced liver toxicity [124]. Zhou *et al.* [12] found that chronic alcohol feeding decreased the expression of miR-122 in liver during the early part of the light/inactive phase of the day (ZT2.5), suggesting that increased circulating levels of miR-122 [124] may indeed be correlated to release from alcohol-damaged hepatocytes [12]. In parallel, alcohol-mediated up-regulation of miR-155, a proinflammatory myeloid cell miRNA, is linked to inflammatory injury in the gut, liver, and brain [123,127,128]. For example, alcohol-mediated increases in miR-155 are associated with the TLR4/NFκB pathway, leading to increased TNF-α production by hepatic Kupffer cells [123]. Importantly, miR-155 knockout mice are protected against alcohol-induced inflammation of the small bowel [127] and neuro-inflammation [128]. Of note, recent work of Curtis *et al.* [129] demonstrated that the molecular clock in myeloid cells may control time-of-day dependent susceptibility to inflammatory stimuli (e.g., LPS) via miR-155, and that miR-155 may feedback and regulate the clock. Based on these exciting findings, future investigations into the role of miRNAs in alcohol-induced tissue injury should consider the potential importance of the time of day and the molecular clock.

In this section, we have highlighted just some of the post-translational and post-transcriptional mechanisms that control the molecular circadian clock and may be involved in the pathogenesis of ALD. Clearly, there are other non-transcriptional mechanisms involving the clock that may also be at play and responsible for alcohol-mediated injury in liver and other organ systems. For example, mounting evidence supports a role of reactive oxygen and nitrogen species (ROS and RNS) in regulating clock function [130]. As these reactive species are implicated in alcohol toxicity, their influence on clock dysregulation in alcohol-exposed tissues should not be ignored. 

## 6. Alcohol and Circadian Clocks

In the alcohol research community, circadian clocks have largely been studied within a neurobiological context, investigating clocks as contributors to alcohol use disorders (e.g., dependence, withdrawal, and addiction) and sleep disorders [131,132,133]. For example, alcohol influences the central SCN clock by altering key functional outputs, such as circadian-driven behaviors (e.g., physical activity) and responsiveness to photic (light) and non-photic (5-HT or glutamate) stimulation [134,135,136,137]. Furthermore, there is evidence pointing to the presence of a temporal pattern in the consumption of alcohol in alcohol-dependent individuals. Danel *et al.* [138] showed that cravings for alcohol in alcohol-dependent patients display a diurnal rhythm that is independent of the subject’s sleep/wake habit. In this study, subjects reported that the time of day at which they consumed their first alcoholic drink was between 9:00 and 11:00 a.m. daily [138]. Importantly, emerging studies indicate a strong link between modulation in alcohol intake and circadian clock disruptions that can occur as a result of environmental and/or genetic factors. For example, circadian disruptions due to environmental factors such as long-term rotating shift work and long working hours in humans [139,140,141,142], single large photoperiod phase advance in rodent models of jet lag [143], and exposure to constant photic conditions in laboratory animals [143,144,145] all significantly increase alcohol intake and preference. Similarly, genetic mutations of circadian clock genes in rodents [146,147] and single nucleotide polymorphisms (SNPs) of clock genes in humans [148,149,150] are associated with increased alcohol consumption. Kovanen *et al.* [151] found several significant gene polymorphisms associated with alcohol dependence and alcohol abuse. For example, they reported suggestive associations of SNPs for *arntl/bmal1*, vasoactive intestinal protein (*vip*), and adenylate cyclase-activating polypeptide 1 (*adcyap1*) with alcohol consumption and *arntl2/bmal2* with alcohol abuse [151]. Interactions of clock genes with other stressors in influencing alcohol consumption and alcohol use disorders have also been investigated. Perreau-Lenz and Spanagel recently highlighted the importance of clock genes (e.g., *per*) in stress-induced alcohol consumption and abuse [152]. Genetic studies using QTL mapping have also identified *per3* as a possible candidate linking the circadian clock, stress, and alcohol [153]. Together, these studies indicate that not only does alcohol consumption alter the SCN circadian clock, but that circadian clock disruption can in turn promote alcohol intake and preference. Notably, these studies identify the SCN circadian clock as a potential target in treating alcohol use disorders.

With this said, the influence alcohol use has on the function of clocks in peripheral tissues, like the liver, and their role in organ pathophysiology remains an underappreciated and understudied area in the field. To date, only a few labs [11,12], including our own [10], have reported on the impact alcohol consumption has on peripheral circadian clocks. Importantly, we were the first to report that chronic alcohol consumption disrupts the function of the circadian clock in the liver and induces circadian desynchrony between the central SCN clock and the peripheral liver clock [10]. This has led us and others to propose that circadian disruption is an important contributor to alcoholic liver injury and possibly alcohol-mediated toxicity to other peripheral organs. In the following sections, we will provide an overview of results collected in this burgeoning new field of alcohol research.

### 6.1. Role of Circadian Clocks in Alcohol-Induced Liver Injury

When peripheral clocks become misaligned with the SCN clock and/or the environment, this leads to a condition of circadian desynchrony, which has been implicated in a variety of diseases, including cancer, diabetes, and cardiovascular disease [154]. Recently, it has been proposed that circadian desynchrony may underpin alcohol-induced tissue injury. Studies from our laboratory show that chronic alcohol consumption disrupts the liver molecular clock without affecting the SCN clock [10]. In this study, 8 week old male C57BL/6J mice and *per2*::Luciferase (Luc) mice were maintained on the Lieber-DeCarli alcohol and control diet regime for five weeks to induce steatosis, oxidative damage, and mitochondrial dysfunction [155]. Mice were maintained under a standard 12:12 L:D cycle. At the end of the feeding period, liver and SCN tissue were collected around the clock at six different time points (ZT 3, 7, 11, 15, 16, and 23; where ZT 0 = lights on and ZT 12 = lights off). Gene expression analysis of 12 core clock component and output genes shows a significant main effect of time for all genes measured in the liver. Chronic alcohol feeding significantly decreases the mesor (*i.e.*, mean expression) of *bmal1*, *clock, cry1*, *cry2*, *per1*, *per2*, and *rev-erba*, as well as the clock-controlled genes *dbp* and *hlf*. Using an *in vitro* model of fatty liver, Tong *et al.* [156] also showed that treatment with the free fatty acid palmitate suppresses the circadian oscillations of *per2*, *rev-erba*, and *dbp*. The amplitude in the rhythm of several clock genes is also significantly reduced in the fatty livers of the ethanol-fed mice as compared to the controls [10]. Interesting, alcohol consumption has no effect on clock gene expression in the SCN, except for a significant phase advance in the peak (timing) of *rev-erba*.

These findings were further validated by using *per2*::Luciferase (Luc) knockin reporter mice, an established model in the chronobiology field. In these mice, the *luc* gene is fused in-frame to the 3' end of the *per2* gene, thus serving as a real-time expression reporter of the clock in all tissues [157]. Organ explants and tissue slices can be kept in culture from SCN and peripheral tissues and show strong and self-sustained circadian *per2*::Luc bioluminescence oscillations for days [10,158]. Using this unique and powerful model, we found that chronic alcohol causes a 3 h phase advance in the liver clock with no effect on the SCN clock [10]. Under normal physiological conditions, the SCN and liver clock have a standard phase relationship of 3–4 h [157]; meaning peak clock gene expression in the liver is delayed about 3–4 h relative to the SCN clock gene expression. It has been proposed that this specific timing relationship is necessary because it allows activity and energy expenditure to occur at a time (determined by the SCN) that aligns with energy metabolism (determined by the liver). In contrast, the SCN-liver phase relationship is significantly decreased by chronic alcohol [10]. Using a similar alcohol feeding protocol, Zhou *et al.* [12] also observed no difference in *per2*::Luc oscillations in SCN from control and alcohol-fed mice, whereas *per2*::Luc rhythms in liver explants from alcohol-fed are anti-phase compared to rhythms measured in control fed *per2*::Luc mice. Based on these findings, we propose that by altering cellular redox state, alcohol preferentially disturbs the liver clock, but not the SCN clock. Importantly, studies suggest that increased NADH phase advances the clock [159], which is supported by advanced clock gene expression in our data [10]. Of note, Zhou *et al.* [12] found that the NAD^+^/NADH rhythms in livers of control and alcohol-fed mice have different phases, and the amplitude of this rhythm is significantly reduced by chronic alcohol feeding. Based on this result, the authors concluded that alteration in the NAD^+^/NADH ratio is likely a key modulator of the circadian clock in alcohol-fed mice. Taken together, these data show that chronic alcohol consumption alters the peripheral clock in the liver and causes desynchrony between the liver clock and the SCN clock.

While these studies have provided compelling evidence for a potential role of clock disruption and desynchrony in alcohol-induced liver injury, only a few investigations have considered the clock in disease pathogenesis from alcohol consumption. Shibata and colleagues [160] reported that female *clock*-mutant mice given alcohol in the drinking water have significantly increased liver weight and hepatic triglyceride content compared to controls. Moreover, alcohol modulates lipid metabolism genes by increasing *acc1* and decreasing *aco* gene expression in livers of *clock-*mutant mice [160]. Alcohol consumption also decreases hepatic expression of *mtp* and induces a slight, but not statistically significant, decrease in *ppara* expression in both *clock-*mutant and wild type mice [160]. From these data, they concluded that disruption of the circadian rhythms associated with the *clock* mutation is an additive risk factor for alcoholic steatosis. In contrast, PER1 knockout mice have a lower level of liver toxicity from acute alcohol administration and lower levels of triglyceride synthesis genes [161].

Studies from our laboratory have also revealed that chronic alcohol feeding significantly disrupts diurnal rhythms in several metabolic genes [10]. For example, diurnal oscillations of the metabolic genes *cpt1a*, *cyp2e1*, *pkc1*, *pdh4*, *ppargc1a*, *ppargc1b*, and *srebp1c* are all lost in the livers of alcohol-fed mice. Moreover, the mesor of *adh1* expression rhythm is significantly decreased by chronic alcohol consumption. While not rhythmic, we also observed that chronic alcohol significantly decreases the overall expression levels in *sirt1*, *ppara*, *adipor1*, *aldh2*, and *fabp1* over the course of the day [10]. Along similar lines, Zhou *et al.* [12] performed a microarray analysis of livers from control and alcohol-fed mice collected at one time point (ZT2.5) and found that alcohol consumption altered the expression of 116 genes, including eight core clock genes. Moreover, almost half of the genes altered in expression by alcohol were identified as clock-controlled genes. Interestingly, the genes up-regulated by alcohol feeding had peak expression between ZT8–ZT16, whereas the genes down-regulated by alcohol had peak expression between ZT20–ZT4 [12]. These results strongly support the concept that alcohol disrupts a temporally coordinated, clock-controlled gene expression program in the liver. Zhou *et al.* [12] also showed that the expression level and phase of several circadian-regulated genes involved in bile acid synthesis are also altered by chronic alcohol feeding [12]. Alcohol consumption increases levels of the lipid metabolism genes *acc1* and *aco1*, as well as, induces a second peak in expression of *fas* [12]. Consistent with these alcohol-mediated alterations in gene expression, Zhou *et al.* [12] demonstrated changes in the levels of several lipid metabolites over the course of the day. For example, hepatic triglyceride levels are elevated in livers of alcohol-fed mice as compared to levels measured in livers from control-fed mice at all time-points of the day, which is consistent with [10]. Hepatic cholesterol and total bile salts levels also showed large time-of-day dependent increases in livers of alcohol-fed mice compared to control-fed mice [12]. In addition to changes in lipid metabolism, alcohol consumption leads to dramatic decreases in hepatic glycogen levels [14,162,163], another important hepatic energy source, which regulates and maintains blood glucose homeostasis. Importantly, our laboratory has reported that depletion in hepatic glycogen levels is likely due to chronic alcohol-induced alterations in the time-of-day dependent rhythms of key components of hepatic glycogen metabolism [14]. Taken together, these studies demonstrate that chronic alcohol consumption disrupts diurnal oscillations in liver metabolic genes that are likely under clock control and strongly supports the hypothesis that chronic alcohol consumption has a significant impact on liver metabolic processes that function in a circadian/diurnal fashion.

The studies described above focused on the impact alcohol has on the liver clock in adults. Interestingly, Farnell *et al.* [164] examined the impact neonatal alcohol exposure had on clock genes (*bmal1*, *per1*, *per2*, *cry1*, and *rev-erba*) in the SCN, cerebellum, and liver. In this study, alcohol was administered on postnatal day 4–9 and tissues were collected when rats were 3 months of age. Alcohol decreases the rhythm of *cry1* expression in the SCN, as well as phase advances oscillations in *per2* expression in the cerebellum and liver. Importantly, this study showed that neonatal alcohol treatment negatively impacts circadian regulation of expression of clock genes in the SCN, as well as peripheral oscillators in other tissues such as the liver and the cerebellum. These results are important as over-nutrition (e.g., high fat diet) early in life disrupts circadian rhythms and impairs liver metabolism [165]. Thus, the impact of early life alcohol exposure on peripheral clocks and disease outcomes is an area in need of increased investigation.

Recent studies [11,166] from Keshavarzian and colleagues have also focused on the potential role of disrupted intestinal clocks in alcohol-induced liver injury. For example, their work has shown that disrupted circadian rhythms by either a genetic model (*clock*Δ19 mutant mice) or environmental means (once weekly repeated 12 h phase shifts in the light-dark cycle) increases gut permeability, which is associated with enhanced alcohol-induced liver injury [11]. Importantly, they showed that alcohol-fed mice subjected to a repeated 12 h light-dark phase shifts have increases in lobular inflammation, liver steatosis, and ballooned hepatocytes as compared to non-shifted alcohol-fed mice and phase shifted control diet mice [11]. In an earlier study, Swanson *et al.* [166] reported that alcohol feeding in rats increases gut permeability and PER2 protein in duodenum and proximal colon. Similarly, siRNA knockdown of *clock* or *per2* decreases alcohol-induced permeability in a Caco-2 cell monolayer system [166]. This finding suggests that alterations in intestinal cell clocks may underpin intestinal permeability and ultimately increase alcohol hepatotoxicity due to higher levels of gut-derived endotoxin in the portal vein circulation. Interestingly, these results show alcohol-mediated increases in clock proteins in the gut, whereas decreases in clock gene and protein expression are detected in liver following alcohol exposure [10,12], suggesting tissue-specific effects of alcohol on the clock.

In summary, these studies demonstrate that chronic alcohol consumption alters diurnal rhythms of clock genes in the liver and gut, as well as several metabolic genes in the liver. It is proposed that these alterations are detrimental and induce a condition of circadian desynchrony or misalignment in metabolic processes, which contribute, in part, to the development and/or progression of alcoholic liver injury. In support of this hypothesis, there is a large and growing body of literature demonstrating a strong interaction between the circadian clock and metabolism. As discussed earlier in this review, the circadian clock mechanism is believed to drive and maintain 24 h rhythms in activity/physiology/metabolism such that organisms/tissues/cells/organelles can easily adapt and respond appropriately to changes in their daily environment. Thus, clocks are believed to be advantageous and provide protection against disease. With this said, aberrant changes in circadian clock gene oscillations are observed in metabolic diseases like obesity and diabetes [167].

Strong support for a role of clocks in metabolic regulation is supported by studies where mutation and/or genetic ablation of clock components perturb rhythmic expression of metabolic genes and energy balance leading to numerous pathological conditions. For example, *clock*Δ19 mutant mice have behavioral, immune, and metabolic disorders due to mutant *clock* in all tissues [34]. Therefore, it is easy to envision that disruption of the clock and downstream clock-regulated metabolic pathways by environmental factors (e.g., chronic alcohol consumption) could have a negative impact on health. A good example has already been shown in the obesity field where a high fat diet attenuates rhythms in peripheral tissue clock genes (liver and adipose) that is associated with weight gain and hepatic steatosis [56]. Taken one step further, it is also easy to speculate that a “mismatch” in the timing of various clock-dependent pathways such as fatty acid synthesis and utilization, glycogen synthesis and breakdown, and antioxidant detoxification pathways, just to name a few, could underpin the pathogenesis of alcoholic liver injury. For example, it is possible that alcohol-mediated steatosis (*i.e.*, fatty liver) is linked to disruption in the normal diurnal oscillations of multiple lipid metabolism components [10,12]. One possible mechanism could involve temporal disruption in the time-of-day accumulation of sterol regulatory element binding protein SREBP-1c in the nucleus due to chronic alcohol-mediated perturbation in the rhythms of the NAMPT/SIRT1/LKB/AMPK signaling axis that regulates SREBP-1c, and most importantly, is under clock control [168]. While still hypothetical, a contribution of the clock in alcoholic steatosis is likely, as a growing literature implicates clock-control over key lipid metabolism components, including SIRT1-AMPK [169] and INSIG-SREBP-1c [170].

### 6.2. One Proposed Model for How Alcohol Disrupts Function of the Liver Clock: Focus on Protein Post-Translational Modifications

Studies from our lab and others demonstrate that chronic alcohol consumption alters time-of-day dependent rhythms in *bmal1* and other core clock genes, various clock-controlled components, many oscillatory metabolic genes, and several physiological/metabolic pathways [10,11,12,14]. However, the question still remains: what are the alcohol-dependent molecular mechanisms and/or metabolic alterations that alter the circadian clock and how do these changes impact, if at all, liver metabolism and liver pathology in the alcohol consumer? One possible mechanism may be how alcohol alters cellular redox (NAD^+^) status and affects redox-dependent PTMs, such as acetylation and ADP-ribosylation. A hypothetical model involving these events is proposed in the following section.

**Figure 4:**
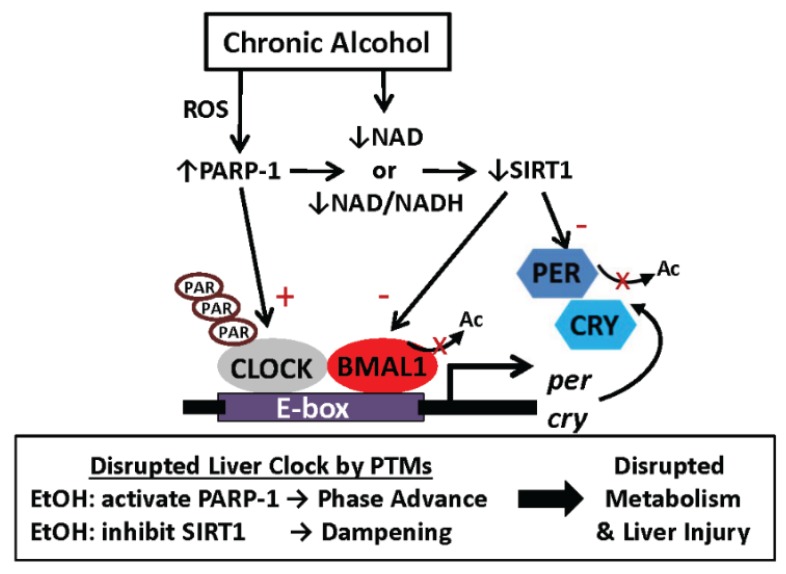
Chronic alcohol consumption disrupts function of the liver clock via changes in PARP-1 and SIRT1 activities. This scheme illustrates one possible hypothetical model to explain how chronic alcohol consumption may perturb the circadian clock mechanism in the liver. The scheme highlights how an alcohol-mediated alteration in the cellular redox state (NAD^+^/NADH) disrupts the liver clock. The NAD^+^-requiring enzymes PARP-1 and SIRT1 are implicated in this mechanism via post-translational modifications of various clock proteins. Ac, acetylation; PAR, ADP-ribosylation; ROS, reactive oxygen species.

As we presented earlier, a link between cellular redox (NAD^+^) metabolism and clock regulation was first provided by Rutter and colleagues in studies showing that DNA binding of the CLOCK-BMAL1 heterodimer can be altered by the absolute levels of pyridine nucleotides and/or the ratio of NAD^+^/NADH [58]. This work shows that NADH increases DNA binding of CLOCK-BMAL1, whereas NAD^+^ decreases DNA binding. Further, NAD^+^ can affect clock activity by influencing the activities of the NAD^+^-dependent enzymes PARP-1 and SIRT1. These enzymes modify clock function by PTM of select clock proteins. Studies have shown that SIRT1 is required for maintenance of robust high-amplitude circadian oscillations of the clock via deacetylation of BMAL1 and PER2 [24,27]. Deacetylation of BMAL1 by SIRT1 modulates CLOCK-BMAL1 dimer binding to target E-boxes [27]. Similarly, SIRT1-dependent deacetylation of PER2, a negative regulator of the clock, triggers PER2 degradation [24]. PARP-1 ADP-ribosylates CLOCK in a time-of-day dependent manner [88]. This action is proposed to influence the timing (phase) of the circadian clock. Importantly, studies show increases in PARP-1 protein and activity (increased ADP-ribosylation) in liver after chronic alcohol consumption [171,172,173]. Because PARP-1 is a major consumer of cellular NAD^+^ [174], we propose that a chronic alcohol-mediated increase in PARP-1 may limit NAD^+^ availability for SIRT1. This outcome, combined with the direct consequence of ethanol metabolism on NAD^+^ metabolism (*i.e.*, a decrease in the NAD^+^/NADH ratio), would likely contribute to decreased SIRT1 activity in liver. In support of this model, we have found that chronic alcohol decreases hepatic *sirt1* mRNA at multiple time points during the day [10] and others have shown decreases SIRT1 activity [85,86] and hyper-acetylation of liver proteins in alcohol-fed mice [175,176]. Taken together, we propose a hypothetical model through which chronic alcohol disrupts the function of the liver clock via perturbations in the normal rhythms of NAD^+^ metabolism and the activities of PARP-1 and SIRT1 throughout the 24 h day. We propose that the chronic alcohol-mediated increase in PARP-1 activity phase advances the liver clock through effects on CLOCK, whereas the alcohol-dependent decrease in SIRT1 activity dampens the amplitude of liver clock rhythms (Figure 4). Consequences of these alcohol-mediated events on the liver clock include disruption and potential desynchronization of tightly-controlled daily oscillations of metabolic pathways (lipid, mitochondrial, antioxidants), leading to liver injury. In parallel, it is well-established that chronic alcohol consumption damages mitochondria [177,178], resulting in ATP depletion in the liver [179,180]. An elevation in the AMP/ATP ratio is predicted to activate AMPK, which in turn directly phosphorylates CRY1; thus, targeting it for degradation [74]. PER2 is also targeted for degradation via an AMPK-mediated activation of CSNK1E, which phosphorylates PER2 [181]. Thus, alcohol-mediated alterations in two key energy sensing metabolites, NAD^+^ and ATP, have the potential to alter the molecular circadian clock machinery through PTM-dependent mechanisms.

### 6.3. Other Possible Mechanisms Responsible for Alcohol-Mediated Alteration in Liver Clock Function

Above we have proposed and described one mechanism through which chronic alcohol consumption might disrupt the molecular circadian clock in the liver. It is clear, however, that there are other mechanisms at play that could disrupt the hepatic clock following chronic alcohol consumption. Several mechanisms were highlighted earlier in the review, and include effects on other post-translational modifications and various post-transcriptional mechanisms (e.g., alterations in miRNAs and the circadian deadenylase Nocturnin). In addition, inflammation and dysregulated cytokine networks are known to be key components in the pathogenesis of alcoholic liver injury [182,183,184]. Of note, various pro-inflammatory cytokines disrupt the function of the molecular clock [185,186,187,188,189]; however, the mechanisms responsible for clock impairment are unknown. This raises the question of whether alcohol-mediated impairment in the hepatic clock may be due, in part, to up-regulation in inflammatory processes. With this in mind, a recent study suggested that the RNA binding protein CIRBP might serve as a unique link between the circadian clock and the immune system [188]. CIRBP alters gene expression by binding to mRNA transcripts thereby affecting stability and/or translation [190,191]. Previous work by Cavadini *et al.* [186] reported that the cytokines tumor necrosis factor-alpha (TNF-α) and interleukin-1 beta (IL-1β) decrease clock gene expression by inhibiting CLOCK-BMAL1 activation of E-box elements in the promoters of various clock genes (e.g., *per1-3*) and clock- controlled genes (e.g., *dbp*, *tef*, and *hlf*). Similarly, Golombek and colleagues showed that lipopolysaccharide (LPS)-induced alterations in clock function are mediated by TNF-α [189] and also dependent on toll-like receptor 4 [192]. More recently, Fontana and colleagues demonstrated that transforming growth factor beta (TGF-β) and TNF-α likely disrupt clock gene expression by inhibiting *cirbp* mRNA expression [188]. While a role of CIRBP is not known in alcohol-induced liver injury, it is possible that alcohol-mediated increases in TNF-α and/or TGF-β might work through CIRBP to impair/decrease clock gene expression in the liver. Evidence to support this concept is provided by work showing a role of CIRBP in alcohol-induced brain inflammation [193]. Future studies examining the impact alcohol has on liver CIRBP expression may yield new insights into the pathobiology of ALD.

### 6.4. Potential Clock-Targeted Treatments for Alcoholic Liver Disease

As recognition of circadian clock disruption in development of human disease has grown, there is a growing list of pharmacological agents that modulate the clock that are being tested in various metabolic diseases. For example, synthetic REV-ERB agonists modulate rhythms in core clock genes and show some promise in improving dyslipidemia [194]. A recent study by Meng *et al.* [195] showed that pharmacological inhibition of CSNK1 increases the amplitude and resynchronization of disrupted circadian clock oscillators. Indeed, the use of CSNK1 inhibitors may be of particular interest as work from Spanagel and colleagues showed that inhibition of CSNK1 with the drug PF-670462 prevents relapse-like alcohol drinking in a rat model [196]. Thus, CSNK1 may be a possible target to not only prevent alcohol drinking, but to also restore clock function and metabolic rhythms in peripheral tissues like the liver; and hence, prevent and/or reverse pathology. Moreover, as chronic alcohol is proposed to perturb NAD^+^ levels and/or rhythms in the liver, agents that restore NAD^+^ may be valuable in treating ALD. Results with NAD^+^ precursors (e.g., nicotinamide riboside) or new generation PARP-1 inhibitors (to increase NAD^+^ bioavailability) show promise in ameliorating various metabolic diseases presumably by preserving NAD^+^ and activating SIRT1 [197,198,199]. However, whether these drugs will impact or normalize clock function is not known, as SIRT1 has effects both upstream and downstream of the circadian clock. Further, it is not clear whether altering NAD^+^ levels alone will affect the clock, as it is the NAD^+^/NADH ratio that it believed to influence clock function [58]. Finally, chrono-therapeutic based studies have already been successful in identifying optimal times for personalized cancer treatment [200]; thus, timed administration of drugs or other interventions may be beneficial for treating ALD patients. Of note, two studies showed that late evening, high carbohydrate-based snacks improve energy metabolism in patients with liver cirrhosis [201,202]. In summary, improved understanding of how alcohol dysregulates the liver clock and downstream clock-controlled pathways will pave the way for identifying new “druggable” targets and the optimal timing of therapies to treat ALD patients. Clearly, this area is wide open for investigation in the alcohol and liver fields.

## 7. Conclusions

The general thesis of this review article is that the function of the circadian clock and clock-regulated mechanisms are negatively impacted by alcohol consumption, resulting in cell dysfunction and tissue pathology. Until recently, the molecular circadian clock has largely been ignored in the study of alcohol-induced liver injury and other peripheral organ systems. To this end, increased understanding into how alcohol disrupts liver and other tissue-specific clocks will shed more light unto the complex mechanisms responsible for alcohol toxicity. Nevertheless, despite this new interest in clocks, full understanding of how clocks and clock disruption contributes to alcohol toxicity in liver and other organs will be complicated by the fact that the clock plays numerous wide-ranging and interconnected roles in metabolism. Investigators will also have to dig deep into understanding not only cell-specific roles of clocks in liver metabolism, but also organ-specific roles of clocks, as alcoholic liver injury is not just a liver disease, but involves dysregulated physiology in multiple organs, including gut, adipose, and brain. Similarly, investigators will have to examine the interaction of alcohol and clock on cells/tissues using systems-based ‘Omics approaches, as discussed herein using the examples of various post-translational and post-transcriptional mechanisms. One should also not lose sight of the possibility that these alcohol-dependent alterations in the clock and/or downstream clock-driven processes in the liver may be adaptive mechanisms that are initiated by the cell/tissue/organism to protect against the metabolic stress and insult induced by chronic alcohol consumption. Consideration of this possibility is warranted when investigating chronic diseases like ALD.

Due to the temporal nature of alcohol consumption and the circadian nature of liver metabolism, the time of day at which alcohol is consumed likely influences tissue injury and disease. In fact, the liver (and other organs) should be thought of as a completely different organ at different times of the day. As such, it is very easy to envision that exposure to a metabolic stress, like alcohol, at different times of the day would have different metabolic and pathological outcomes. Indeed, the importance of time on pathological outcomes was recently highlighted in studies showing that the time-of-day of fat intake altered key cardiometabolic parameters. Consumption of a high fat diet at the end of the active phase of the day (in mice) increases adiposity, dyslipidemia, and cardiac dysfunction [55] and disrupts hepatic metabolism [203]. Based on this, future studies should investigate time as an independent risk factor for alcoholic liver injury. Moreover, there may be “windows” during the day when the circadian clock is more vulnerable to perturbation by alcohol consumption. Increased understanding of the importance of “time” within the etiology of alcohol toxicity may also reveal more efficacious temporal windows for therapeutic interventions in treating ALD patients. This is critical as drugs used to treat ALD may target circadian-dependent metabolic pathways. Failures in drug trials for treating ALD might be related to the fact that the time of drug delivery did not match that of the clock and/or the clock-controlled drug targets. However, due to the ubiquitous presence of clocks in most cells of the body and their fundamental role in regulating a plethora of behavioral and physiological processes it will be very difficult to target a specific pathway implicated in ALD without having off-target effects.

In summary, we and others have shown that alcohol dampens and disrupts clock rhythms in the liver, and desynchronizes the liver clock from the primary, central SCN clock [10,12]. Therefore, drugs or interventions that boost, normalize, and/or resynchronize clock rhythms have high potential for treating ALD. In the end, addressing these scientific questions and others will hopefully identify the clock as a fundamental component of alcoholic liver injury and open the door for new chrono-therapeutic strategies to treat individuals with ALD and other related hepatic pathologies.
